# Pathological complete response after cisplatin neoadjuvant therapy is associated with the downregulation of DNA repair genes in *BRCA1*-associated triple-negative breast cancers

**DOI:** 10.18632/oncotarget.11900

**Published:** 2016-09-08

**Authors:** Pawel Domagala, Jolanta Hybiak, Janusz Rys, Tomasz Byrski, Cezary Cybulski, Jan Lubinski

**Affiliations:** ^1^ Department of Pathology, Pomeranian Medical University, Szczecin, Poland; ^2^ Department of Tumor Pathology, Maria Sklodowska-Curie Memorial Cancer Centre & Institute of Oncology, Krakow Branch, Krakow, Poland; ^3^ Department of Oncology, Pomeranian Medical University, Szczecin, Poland; ^4^ Department of Genetics and Pathology, International Hereditary Cancer Center, Pomeranian Medical University, Szczecin, Poland

**Keywords:** breast cancer, triple-negative, DNA repair, cisplatin, BRCA1

## Abstract

Pathologic complete response (pCR) after neoadjuvant chemotherapy is considered a suitable surrogate marker of treatment efficacy in patients with triple-negative breast cancers (TNBCs). However, the molecular mechanisms underlying pCR as a result of such treatment remain obscure. Using real-time PCR arrays we compared the expression levels of 120 genes involved in the main mechanisms of DNA repair in 43 pretreatment biopsies of *BRCA1*-associated TNBCs exhibiting pCR and no pathological complete response (non-pCR) after neoadjuvant chemotherapy with cisplatin. Altogether, 25 genes were significantly differentially expressed between tumors exhibiting pCR and non-pCR, and these genes were downregulated in the pCR group compared to the non-pCR group. A difference in expression level greater than 1.5-fold was detected for nine genes: *MGMT*, *ERCC4*, *FANCB*, *UBA1*, *XRCC5*, *XPA*, *XPC*, *PARP3*, and *RPA1*. The non-homologous end joining and nucleotide excision repair pathways of DNA repair showed the most significant relevance. Expression profile of DNA repair genes associated with pCR was different in the node-positive (20 genes with fold change >1.5) and node-negative (only 3 genes) subgroups. Although *BRCA1* germline mutations are the principal defects in *BRCA1*-associated TNBC, our results indicate that the additional downregulation of other genes engaged in major pathways of DNA repair may play a decisive role in the pathological response of these tumors to cisplatin neoadjuvant chemotherapy. The results suggest that patients with node-positive *BRCA1*-associated TNBCs that do not exhibit pCR after cisplatin neoadjuvant chemotherapy may be candidates for subsequent therapy with PARP inhibitors, whereas UBA1 may be a potential therapeutic target in node-negative subgroup.

## INTRODUCTION

Triple-negative breast cancer (TNBC) is defined by the lack of immunohistochemical expression of the estrogen receptor (ER) and progesterone receptor (PR) and the absence of human epidermal growth factor receptor 2 (HER-2) overexpression. TNBC accounts for 15-20% of breast cancer cases [1]. Patients with TNBC show poor recurrence-free and overall survival and a high risk of relapse, and there are few treatment options available for them [2, 3]. TNBCs constitute approximately 80% of *BRCA1*-associated breast cancers [4]. TNBC is a heterogeneous disease so various TNBC subgroups may show differential responses to treatment [5]. In particular, these tumors are known to be sensitive to inter-strand cross-linking agents, including platinum analogs because they carry a defect in the DNA double-strand break (DSB) repair pathway. They are also sensitive to poly(ADP-ribose) polymerase (PARP) inhibitors in the mechanism of synthetic lethality [6].

Recently, there has been a resurgence of interest in platinum-based chemotherapy [7] including the use of platinum in patients with *BRCA1*-associated breast cancer which is known for its germline deficiency in DNA damage repair via homologous recombination (HR) [8]. Cisplatin is an alkylating agent used in the treatment of various cancers such as those of the ovary, testis, lung, head and neck. Cisplatin induces DNA adducts and intrastrand and interstrand crosslinks, which leads to single- and double-strand DNA breaks, the activation of the DNA damage response, and finally to the apoptosis of tumor cells [9]. Several major DNA repair pathways (mechanisms) are known, including nucleotide excision repair (NER), base excision repair (BER), HR, non-homologous end joining (NHEJ), mismatch repair (MMR), and the Fanconi anemia pathway (FAP) [10]. Once the platinum is bound to the DNA of tumor cells, the cells activate various pathways of DNA repair, predominantly NER, to survive [7, 11]. There is, however, accumulating evidence from studies on cell lines that sensitivity or hypersensitivity to cisplatin is associated with an inherent (constitutive) reduced DNA repair capacity in response to platinum-DNA adducts (reviewed in [7]). Specifically, low constitutive NER capacity and low levels of XPA, XPF and ERCC1 (NER proteins) have been reported in cisplatin-hypersensitive testicular cancer cells compared to other tumor types resistant to cisplatin [12].

Neoadjuvant chemotherapy with cisplatin has been applied in patients with *BRCA1*-associated breast cancer [13] and TNBC [14]; however, the outcomes of cisplatin-based neoadjuvant chemotherapy are heterogeneous. Approximately 60% of patients with *BRCA1*-associated breast cancer treated with neoadjuvant chemotherapy with cisplatin obtain a pathologic complete response (pCR) whereas a sizable percentage of such patients do not experience pathological complete response (non-pCR). The pCR to neoadjuvant chemotherapy, defined as ypT0 ypN0, is regarded as an independent predictive factor of favorable clinical outcome [15–17] and is considered a suitable surrogate marker of treatment efficacy for patients with TNBCs [15, 18, 19]. The dramatic positive therapeutic effect (pCR) of cisplatin treatment in patients carrying *BRCA1*-associated cancers has been generally attributed to germline *BRCA1* mutations and to the dysfunction of DNA damage repair by HR. However, it is not known whether other important pathways of DNA damage repair operating in cancer cells contribute to and influence the pathologic response in a cisplatin neoadjuvant chemotherapy setting in *BRCA1*-associated TNBCs.

The aim of this report was to compare the expression profiles of genes associated with major pathways of DNA damage repair in pretreatment biopsies of *BRCA1*-associated TNBCs exhibiting a pCR *vs.* non-pCR after neoadjuvant chemotherapy with cisplatin. We tested the hypothesis that *BRCA1*-associated TNBCs exhibiting pCR and non-pCR may differ in the expression of DNA damage repair genes before the onset of neoadjuvant chemotherapy with cisplatin, which could explain the differences in pathologic response. To this end, using real-time quantitative polymerase chain reaction (qPCR), we determined the expression levels of 120 genes representing all six main mechanisms of DNA damage repair (NER, BER, NHEJ, MMR, HR, and FAP) in pretreatment biopsies of *BRCA1*-associated TNBCs.

## RESULTS

### Cisplatin sensitivity of *BRCA1*-associated TNBCs depends on the expression of DNA repair genes in pretreatment biopsies

**Figure 1:**
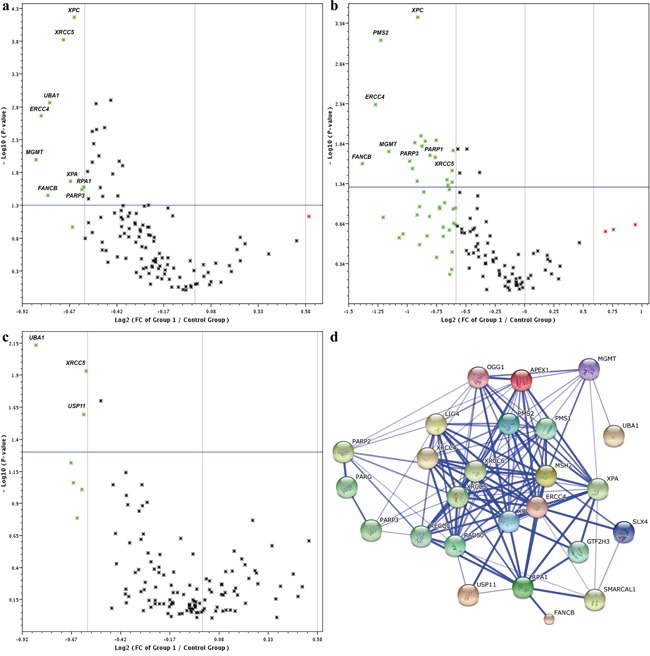
Volcano plots of 120 DNA repair genes between pCR and non-pCR groups in a. all *BRCA1*-associated TNBC patients, b. the lymph node-positive group, and c. the lymph node-negative group Log2 FC (fold changes; x-axis) in gene expression are plotted against *P*-values (y-axis). Green symbols in the Volcano plot above the blue line (*P*<0.05) readily identify downregulated genes with differences greater than 1.5-fold. **d.** STRING molecular network of proteins coded by cisplatin-responsive genes. Gene symbols corresponding to the set of 25 downregulated genes in the pCR group (Table 1) were imported into STRING and the complex protein-protein interaction network is shown on the confidence view of STRING. Proteins coded by the 25 genes form 127 functional interactions. Stronger associations are represented by thicker lines. This network highlights multiple relationships in which proteins coded by DNA repair genes involved in cisplatin sensitivity function and illustrates extensive crosstalk among DNA repair pathways.

The expression profiles of 120 genes involved in DNA damage repair were analyzed using real-time PCR arrays in pretreatment biopsies of *BRCA1*-associated TNBCs. The fold change in the expression of all genes in the pCR group *vs.* the non-pCR group is shown in Figure 1a and Table 1. Altogether 25 genes were significantly differentially expressed between the groups and these genes were downregulated in the pCR group compared with the non-pCR group. A difference in expression level greater than 1.5-fold was detected for nine of these genes: *MGMT* (*P*=0.01), *ERCC4* (*P*=0.002), *FANCB* (*P*=0.03), *UBA1* (*P*=0.001), *XRCC5* (*P*=0.0002), *XPA* (*P*=0.02), *XPC* (*P*=0.00007), *PARP3* (*P*=0.03), and *RPA1* (*P*=0.03) (Table 1). Four of the nine genes (*ERCC4*, *XPA*, *XPC*, and *RPA1*) are predominantly involved in the NER pathway and *XRCC5* is involved in the NHEJ pathway.

**Table 1:** Genes downregulated in pCR group compared with non-pCR group (all patients)

Gene name	Pathways^*^	Fold-change	*P*-value
*MGMT*	DNA DR	-1.79	0.01
*ERCC4*	NER, HDR, FAP	-1.76	0.002
*FANCB*	FAP	-1.72	0.03
*UBA1*	DNA DSB Response	-1.71	0.001
*XRCC5*	NHEJ	-1.62	0.0002
*XPA*	NER	-1.58	0.02
*XPC*	NER	-1.56	0.00007
*PARP3*	BER	-1.52	0.03
*RPA1*	NER, HDR, FAP, MMR	-1.51	0.03
*LIG4*	NHEJ	-1.49	0.04
*PMS2*	MMR	-1.48	0.02
*PMS1*	MMR	-1.46	0.004
*MSH2*	MMR	-1.46	0.01
*SMARCAL1*	NHEJ	-1.45	0.006
*OGG1*	BER	-1.44	0.002
*SLX4*	HDR, FAP	-1.43	0.004
*GTF2H3*	NER	-1.43	0.009
*USP11*	HDR	-1.41	0.04
*PARG*	BER	-1.40	0.04
*RAD50*	HDR, NHEJ	-1.39	0.003
*APEX1*	BER	-1.36	0.001
*XRCC4*	NHEJ	-1.35	0.02
*PARP2*	BER	-1.33	0.04
*RECQL*	NER, MMR, NHEJ	-1.32	0.01
*XRCC6*	NHEJ	-1.32	0.01

* Genes were assigned to DNA repair pathways according to REACTOME DNA repair pathway hierarchy: base excision repair (BER); nucleotide excision repair (NER); mismatch repair (MMR); Fanconi anemia pathway (FAP); DNA damage reversal (DNA DR); DNA double-strand break repair (DNA DSB): DNA DSB response, homology directed repair (HDR), nonhomologous end-joining (NHEJ).

Pathway identification by KEGG analysis showed the involvement of differentially expressed DNA repair genes in five relevant pathways. Five of these genes (*ERCC4*, *XPA*, *XPC*, *RPA1*, and *GTF2H3*) were involved in the NER pathway, five genes in NHEJ (*XRCC4*, *XRCC5*, *XRCC6*, *RAD50*, and *LIG4*), five genes in FAP (*FANCB, RPA1, ERCC4, PMS2*, and *SLX4*), four genes in BER (*PARP2, PARP3, OGG1*, and *APEX1*), and three genes (*MSH2, PMS2*, and *RPA1*) in MMR pathway. Differentially expressed pathways and genes and their *P* values are shown in Table 2. Differentially expressed genes were submitted to STRING 10 to detect possible protein-protein interactions and the results showed that 127 experimentally proven protein-protein interactions were formed by 25 genes (Figure 1d).

**Table 2:** DNA repair pathways identified by KEGG analysis of differentially expressed genes

Pathway	Genes	*P*-value
All		
Nonhomologous end-joining	*XRCC4, XRCC5, XRCC6, RAD50, LIG4*	6.5e-10
Nucleotide excision repair	*ERCC4, XPA, XPC, RPA1, GTF2H3*	3.4e-7
Fanconi anemia pathway	*FANCB, RPA1, ERCC4, PMS2, SLX4*	3.9e-7
Base excision repair	*PARP2, PARP3, OGG1, APEX1*	5.0e-6
Mismatch repair	*MSH2, PMS2, RPA1*	1.6e-4
Lymph node-positive		
Nonhomologous end-joining	*XRCC5, XRCC6, RAD50, LIG4, MRE11A*	4.1e-10
Fanconi anemia pathway	*FANCB, ERCC4, SLX4, REV1, PMS2*	3.7e-7
Nucleotide excision repair	*ERCC4, ERCC5, XPC, GTF2H3*	1.8e-5
Homologous recombination	*RAD50, MRE11A, SHFM1*	2.6e-4
Base excision repair	*PARP1, PARP3, APEX1*	3.8e-4

### Expression profile of DNA repair genes associated with pCR is different in pretreatment biopsies of the node-positive and the node-negative *BRCA1*-associated TNBCs

Next, we focused on pretreatment biopsies of patients with clinically positive *vs.* negative axillary lymph nodes. A total of 24 significantly differentially expressed genes were found in the node-positive subgroup, and only four were found – in the node-negative subgroup (Figure 1b and 1c, Table 3). As shown in Table 3, the expression profile of DNA repair genes associated with pCR was different in the node-positive and the node-negative subgroups of *BRCA1*-associated TNBCs.

**Table 3:** Genes downregulated in the pCR group compared with non-pCR group in lymph node-positive and lymph node-negative patients

Lymph node-positive			Lymph node-negative		
Gene	Fold-change	*P*-value	Gene	Fold-change	*P*-value
*FANCB*	-2.61	0.03	*UBA1*	-1.80	0.007
*ERCC4*	-2.42	0.005	*USP11*	-1.52	0.02
*PMS2*	-2.35	0.0007	*XRCC5*	-1.51	0.01
*MGMT*	-2.23	0.02	*OGG1*	-1.43	0.02
*PARP3*	-1.98	0.02			
*BCCIP*	-1.94	0.03			
*ERCC5*	-1.92	0.01			
*LIG4*	-1.89	0.04			
*XPC*	-1.88	0.0004			
*SHFM1*	-1.85	0.01			
*MAPKAPK2*	-1.84	0.02			
*RAD50*	-1.80	0.01			
*UBA1*	-1.75	0.02			
*PARP1*	-1.70	0.02			
*PMS1*	-1.69	0.01			
*SLX4*	-1.58	0.04			
*GTF2H3*	-1.58	0.04			
*XRCC5*	-1.54	0.03			
*REV1*	-1.53	0.04			
*MRE11A*	-1.53	0.02			
*SMARCAL1*	-1.48	0.02			
*APEX1*	-1.47	0.03			
*SPIDR*	-1.41	0.02			
*XRCC6*	-1.34	0.02			

In the node-positive subgroup, a greater than two-fold difference in expression levels between the pCR and non-pCR groups was seen for four genes: *FANCB* (*P*=0.03), *ERCC4* (*P*=0.005), *PMS2* (*P*=0.0007), and *MGMT* (*P*=0.02). Sixteen genes showed a difference in expression levels greater than 1.5-fold. All these genes were downregulated in the group with pCR compared with non-pCR. Conversely, these genes were upregulated in the non-pCR group including *PARP1* (1.7-fold change, *P*=0.02) and *PARP3* (1.98-fold change, *P*=0.02).

Altogether, pathway identification by KEGG analysis in the node-positive subgroup showed the involvement of differentially expressed DNA repair genes in five relevant pathways. Five of these genes (*XRCC5*, *XRCC6*, *RAD50*, *LIG4*, and *MRE11A*) were involved in NHEJ, five genes (*FANCB*, *ERCC4*, *SLX4*, *REV1*, and *PMS2*) in FAP, four genes (*ERCC4*, *ERCC5*, *XPC*, and *GTF2H3*) in NER, three genes (*RAD50, MRE11A*, and *SHFM1*) in HR, and three genes (*PARP1, PARP3*, and *APEX1*) in BER pathways. Differentially expressed genes, pathways and *P* values are shown in Table 2. Thus, the major downregulated pathways in node-positive TNBCs that underwent pCR were NHEJ, FAP, NER, HR, and BER.

In the node-negative subgroup, only four genes were significantly differentially expressed between the pCR and non-pCR groups (Figure 1c, Table 3). A 1.8-fold difference was detected for *UBA1* (P=0.007), and for the remaining three genes (*USP11*, *XRCC5*, and *OGG1*) the differences ranged from 1.5-fold to 1.4-fold. *UBA1* is involved in the DNA DSB response, *XRCC5* and *USP11* are involved in DSB repair (by NHEJ and HR, respectively), and *OGG1* is involved in the BER pathway. In particular, in this subgroup, genes involved in the NER pathway were not differentially expressed, and no DNA repair pathway was identified by KEGG analysis to be significantly relevant.

## DISCUSSION

Deficiency in DNA damage repair is commonly found in many cancers [20, 21], however, it remains unclear whether and how this defect may influence the pathological response after cisplatin neoadjuvant chemotherapy in *BRCA1*-associated TNBCs. BRCA1 is part of the BRCA/Fanconi anemia DNA repair pathway, is engaged in the repair of DNA DSBs, stalled replication forks, and DNA crosslinks by HR [22]. Indeed, BRCA1-deficient cells are highly sensitive to cisplatin *in vitro* [23] because they cannot use HR to efficiently repair DNA DSBs; therefore, they use alternative, error-prone DNA repair systems (e.g., NHEJ). In addition, complex DNA cisplatin-double strand break lesions directly impair cellular NHEJ [24]. As a result, chromosomal instability (mutations, translocations) increases and when it exceeds the ability of cellular DNA damage response mechanisms to repair the damage, BRCA1-incompetent tumor cells are directed on the pathway to apoptosis. However, although all tumors in the present study were *BRCA1*-associated, and in theory should respond to cisplatin with the apoptosis of tumor cells, 40% of them did not exhibit pCR after neoadjuvant chemotherapy with cisplatin. Our results showed that 25 genes involved in five different pathways of DNA damage repair were significantly differentially expressed between the pCR and non-pCR groups in pretreatment biopsies. Thus, *BRCA1*-associated TNBCs were heterogeneous with regard to the expression of DNA damage repair genes and the downregulation of the set of these genes before the onset of neoadjuvant chemotherapy with cisplatin may partially explain the differences in the pathologic response to treatment. To the best of our knowledge, this study is the first to identify a set of differentially expressed DNA damage repair genes between tumors undergoing pCR and non-pCR after neoadjuvant chemotherapy with cisplatin in pretreatment biopsies of *BRCA*-associated TNBCs.

Cisplatin treatment triggers DNA damage recognition and repair predominantly through NER mechanisms [7, 25]. In the present report, we document that the cisplatin sensitivity (pCR) of *BRCA1*-associated TNBCs was associated with downregulation of the NER pathway of DNA repair in pretreatment biopsies. Compared to the non-pCR group, the reduced expression of *XPC*, *XPA*, *ERCC4*, *GTF2H3*, and other DNA repair genes involved in NER was observed. Because NER is the principal mechanism used to remove cisplatin lesions from DNA, such tumors would have intrinsic hypersensitivity to cisplatin (pCR) due to their reduced ability to repair DNA-platinum adducts and intra- and interstrand crosslinks. These findings are consistent with reported hypersensitivity to cisplatin of testicular cancer cell lines as a result of reduced levels of DNA repair proteins [26–28], especially NER proteins (*XPA*, *XPF*, and *ERCC1*) [12]. Furthermore, interstrand crosslinks repair requires cooperation between the FA and NER pathways [25]. In the present report, *FANCB* and three other genes cooperating with the FA pathway were found to be downregulated in cisplatin-sensitive tumors. Thus, our results point to an important role of the reduced expression of DNA repair genes involved in the NER and FA pathways in the sensitivity of *BRCA1*-associated TNBCs to neoadjuvant chemotherapy with cisplatin.

Furthermore, our results indicate that pCR of *BRCA1*-associated TNBCs after neoadjuvant chemotherapy with cisplatin cannot be attributed to only the inactivation of one pathway of DNA damage repair (i.e., HR due to germline *BRCA1* mutations, as this was present in both pCR and non-pCR groups). Instead, this response depends on decreased DNA repair capacity due to the constitutive (intrinsic) pretreatment downregulation of genes involved in additional important pathways of DNA damage repair (i.e., NHEJ, NER, FAP, BER, MMR, and MGMT mechanisms). Thus, *BRCA1*-associated TNBCs exhibiting pCR seem to be ideal examples of a state of readiness for “intrinsic” synthetic lethality triggered by cisplatin. Not only HR and BER are impaired (as in *BRCA1*-associated tumors treated by PARP inhibitors); additionally, several other important DNA damage repair pathways, which are needed to repair DNA damage induced by cisplatin, are intrinsically downregulated so that tumor cells cannot efficiently cope with the overload of DNA damage inflicted by this drug. Thus, pCR of *BRCA1*-associated TNBCs to cisplatin not only is the result of germline *BRCA1* mutations but seems to be the effect of rather complex multigene mechanisms.

Our results highlight multiple relationships in which DNA repair genes involved in cisplatin sensitivity function. Indeed, accumulating evidence from cell line studies indicates the existence of extensive crosstalk among DNA repair pathways. Several DNA damage repair genes significantly downregulated in *BRCA1*-associated TNBCs in the pCR subgroup, (e.g., *ERCC4, RPA1*) are known to be involved in multiple pathways of DNA damage repair [25]. For example, *ERCC4* codes for the DNA repair endonuclease XPF, which, together with ERCC1, builds the enzyme complex (ERCC1-XPF) that is engaged in NER and the repair of DSBs and interstrand crosslinks.

Although several cell line studies have suggested an association between certain DNA damage response genes or proteins and cisplatin resistance [29], the contribution of the expression of DNA damage repair genes to cisplatin sensitivity/resistance in *BRCA1*-associated TNBCs is not fully understood. In the present study, the non-pCR group of *BRCA1*-associated TNBCs, in comparison to their sensitive counterparts exhibited increased expression of the set of DNA damage repair genes involved in the main DNA repair pathways, such as NER, NHEJ, and BER, suggesting an increased capacity for DNA repair. This result is in line with those of studies suggesting that increased NER, NHEJ, and HR are the most important mechanisms for cisplatin resistance in tumor cell lines [30, 31]. Similarly, cisplatin-resistant human ovarian cancer cell lines have been shown to have increased DNA repair capacity in comparison to their sensitive counterparts [32].

The pCR rate in node-positive *BRCA1* mutation carriers treated with cisplatin neoadjuvant chemotherapy has been reported to be 39%, whereas the rate in node-negative patients is 71% [13]. Interestingly, in the present study, in the node-positive and node-negative *BRCA1*-associated TNBCs, different DNA repair pathways were downregulated in pretreatment biopsies of patients exhibiting pCR to cisplatin. In the group of node-positive patients the major downregulated pathways were NHEJ, FAP, and NER whereas in the node-negative group, no pathway was found to be relevant. Downregulation of *FANCB* (2.61-fold change) is of interest because little is known about the expression of this member of the FA family in general and in cisplatin sensitivity in particular. It has been suggested that the inhibition of the FA pathway represents a possible route to sensitization of tumors to DNA crosslinking drugs such as cisplatin [33].

Patients with TNBC and remaining residual disease (non-pCR) after neoadjuvant chemotherapy are known to have a high risk of early recurrence and a dismal prognosis, however, there is currently no effective treatment for these patients [14, 34]. In this context, our results suggest that node-positive patients with *BRCA1*-associated TNBCs who experienced a non-pCR after neoadjuvant chemotherapy with cisplatin seem to be good candidates for subsequent therapy with PARP inhibitors because in this group, *PARP3* and *PARP1* (genes involved in BER) were significantly upregulated in pretreatment biopsies.

In the node-negative group, neither the NER nor BER pathway was found to be relevant to the pathologic response after cisplatin neoadjuvant chemotherapy. This finding suggests that the likelihood that patients with node-negative *BRCA1*-associated TNBCs who do not exhibit pCR after cisplatin neoadjuvant chemotherapy would experience any benefit from additional treatment with PARP inhibitors is very low. However, our data suggest UBA1 (Ubiquitin-Activating Enzyme E1 Homolog A) as a potential therapeutic target in patients with node-negative *BRCA1*-associated TNBCs who did not exhibit pCR after cisplatin neoadjuvant chemotherapy. Of four downregulated genes of the node-negative *BRCA1*-associated TNBCs exhibiting pCR, *UBA1* exhibited the most reduced expression (1.8-fold) compared to the non-pCR subgroup. *UBA1* is required for timely cellular response to DNA damage. It has been identified as candidate olaparib sensitivity gene [35]. The protein product of this gene is involved in the recruitment of TP53BP1 and BRCA1 at DNA damage sites [36]. UBA1 protein represents the E1 component of the ubiquitylation cascade and the ubiquitylation of proteins is one of the most important processes operating within the DNA damage repair network [36]. In this respect, it is of interest that deubiquitinase inhibition has been proposed as a cancer therapeutic strategy [37]. Indeed, small molecule inhibitors targeting E1 have recently been reported [38, 39].

Recently, significant effort has been directed at identifying breast cancers exhibiting so-called BRCAness. In the recent review the following definition has been proposed: “BRCAness is a phenocopy of *BRCA1* or *BRCA2* mutation; it describes the situation in which an homologous recombination repair defect exists in a tumour in the absence of a germline *BRCA1* or *BRCA2* mutation” [40]. Thus, BRCAness refers to phenotypic characteristics that some sporadic breast cancers especially TNBCs may share with *BRCA*-associated breast cancers. The expectation is that these tumors would react to therapy similarly to *BRCA1*-associated breast cancers including sensitivity to cisplatin or PARP inhibitors. However, it has been reported that *BRCA1*-associated breast cancers are not homogeneous in respect of their response to neoadjuvant chemotherapy [13], and in the present report, we point to DNA damage repair as the molecular background of this heterogeneity. Therefore, our results generate the hypothesis that true BRCAness represents a phenotype of sporadic breast cancer that is defined by HR deficiency and simultaneous downregulation of important genes involved in additional pathways of DNA repair in tumor cells. Furthermore, depending on the type of chemotherapeutic drug, that is, the type of DNA damage inflicted by the drug, the downregulation of different DNA repair genes may be required to achieve pCR. Which DNA repair genes should be downregulated obligatorily and the best combination of downregulated genes to achieve pCR require further study. To take therapeutic advantage of such defined BRCAness, the development of clinically useful tests that can assess the proficiency of DNA repair in pretreatment biopsies is necessary. The importance of evaluation of DNA damage repair competence for prediction of breast cancer sensitivity to neoadjuvant chemotherapy has been suggested based on immunohistochemical assessment of nuclear focus formation of four DNA damage repair proteins [41].

This study provides novel insights into the potential role of various DNA repair pathways in relation to sensitivity to platinum-based neoadjuvant chemotherapy in patients with *BRCA1*-associated TNBC. These results may also have implications for treatment of patients with non-*BRCA*-related TNBC. Although the study subgroups are relatively small however, the results are statistically significant and supported by literature data from basic research on tumor cell lines. Moreover, the study was based on clinical tissue specimens whereas the majority of reports on mechanisms of sensitivity to cisplatin have been based on cell lines [12, 24–28].

In conclusion, our results indicate that *BRCA1*-associated TNBCs exhibiting pCR are characterized by a lower DNA-repair capability due to not only the germline inactivation of *BRCA1* but also the simultaneous downregulation of other important DNA damage repair genes engaged in several additional pathways of DNA repair. Thus, pCR is achieved because *BRCA1*-associated TNBCs are characterized by their readiness for “intrinsic” synthetic lethality triggered by cisplatin. From the point of view of the proficiency of DNA damage repair, *BRCA1*-associated TNBCs are not a homogenous group; therefore, they should not be regarded as such when patients with this type of cancer are enrolled in clinical trials of neoadjuvant chemotherapy with cisplatin or PARP inhibitors. Therefore, our results may have relevance in the proper selection of patients with *BRCA1*-associated TNBCs (and TNBCs in general) for clinical trials that assess the efficacy of neoadjuvant chemotherapy with cisplatin, other chemotherapeutic drugs, or PARP inhibitors. Finally, our results suggest that one way to improve the results of neoadjuvant chemotherapy of patients with *BRCA1*-associated TNBCs who do not exhibit pCR to cisplatin may be to search for inhibitors of functionally important proteins coded by DNA repair genes that are upregulated in the non-pCR group.

## MATERIALS AND METHODS

### Patients

This study included 43 unselected TNBC patients with *BRCA1* germline mutation who had received neoadjuvant chemotherapy with cisplatin from 2008 to 2014 in Szczecin and Krakow. Patients were eligible for the study if they had a new diagnosis of clinical stage I-III triple-negative invasive breast cancer, pathologically confirmed by core biopsy and core biopsy tissue samples were available. Patients with a previous diagnosis of cancer in the contralateral breast or ovary were also eligible. Patients who had received previous neoadjuvant chemotherapy for the current diagnosis were not eligible. The tumor pathology was reviewed on a core biopsy to confirm both the diagnosis and a tumor content of at least 70%. Pretreatment lymph node status was assessed as described previously [8]. Evaluation of ER, PR, and HER2 immunohistochemistry was performed on the core biopsy prior to starting chemotherapy. *BRCA1* testing was conducted as described previously [42]. The clinicopathologic characteristics of the patients are shown in Table 4.

**Table 4:** Baseline clinical characteristics of the 43 patients with *BRCA1*-associated triple-negative breast cancer

Characteristic	n	%
Age		
Mean	44.5	
Range (years)	28-66	
Genotype of *BRCA1* mutation		
5382insC	35	81.4
C61G	7	16.3
4153delA	1	2.3
Treating hospital		
Szczecin	34	79.1
Krakow	9	20.9
Clinical tumor size		
cT1	17	39.5
cT2	23	53.5
cT3	3	7.0
Clinical nodal status		
Negative	27	62.8
Positive	16	37.2
Pathologic response		
Complete	26	60.5
Non-complete	17	39.5

Patients were treated with cisplatin chemotherapy at a dose of 75 mg/m^2^ every 3 weeks for four cycles. Each cycle lasted for 21 days. Detailed information about the treatment of patients was described previously [13]. After the completion of cisplatin chemotherapy, all patients were treated with mastectomy and axillary lymph node dissection. Sampling of post-treatment mastectomy specimens was similar in both groups (pCR vs. non-pCR) according to one standard protocol. The specimens were evaluated for chemotherapeutic response. All patients provided written informed consent to participate in the trial. The trial is registered on ClinicalTrials.gov (study number NCT01630226) and the current study was approved by the Research Ethics Review Board of the Pomeranian Medical University.

### Response criteria

The primary endpoint of this study was pCR. The initial clinical stage and post- neoadjuvant chemotherapy pathologic stage were evaluated, based on the AJCC 7th edition [17]. Pathologic response was considered complete if there was no evidence of residual invasive cancer in the breast and the lymph nodes. If there was evidence of breast carcinoma *in situ* but no evidence of invasive disease, this was still considered pCR [13, 17]. All other responses were included in non-pCR group.

### Identification of genes involved in DNA repair pathways

Based on a literature review we chose a set of 120 DNA repair genes with documented roles in the following DNA repair pathways: BER, NER, MMR, FAP, DNA damage bypass (DNA DB), DNA damage reversal (DNA DR), homology directed repair (HDR), and NHEJ. Special attention was paid to genes described as associated with sensitivity to PARP inhibition [43–46]. The list of genes analyzed in this study are shown in Supplementary Table S1.

### RNA isolation

Seven serial 7-μm-thick sections were cut from each core biopsy fixed in 10% neutral buffered formalin and embedded in paraffin (FFPE). Total RNA was extracted using the RNeasy FFPE Kit (Qiagen, Hilden, Germany). Tissue was digested overnight at 56°C with 10 μl proteinase K and 150 μl PKD buffer. To reverse formaldehyde modifications, samples were incubated at 80°C for 15 min. The lysate was centrifuged for 5 min at 14000 rpm, and the supernatant was transferred to new 1.5 μl tubes without disturbing the debris. gDNA was removed by incubation of the lysate with 10 μl DNase I and 16 μl DNase Booster Buffer at room temperature for 15 min. Then, the lysate was transferred to a new 1.5 ml microcentrifuge tube, and 320 μl RBC buffer and 720 μl ethanol (100%) were added. 700 μl of the sample were transferred to an RNeasy MinElute spin column placed in a 2 ml collection tube and centrifuged for 15 sec at 10,000 rpm; the flow-through was discarded. This step was repeated. To wash the spin column membrane 500 μl RPE buffer was added and centrifuged for 15 sec at 10,000 rpm, and the flow-through was discarded. Next, 500 μl RPE buffer was added and centrifuged for 2 min at 10,000 rpm. To dry the spin column membrane, the column was placed in a new 2 ml collection tube and centrifuged for 5 min at full speed. For elution, 14 μl of RNase-free water was used. All steps of the procedure were conducted under RNase-free conditions with caution to avoid contamination.

The concentration of total RNA (ng/μl) was determined at an absorbance of 260 nm (A260) using a NanoDrop 2000 spectrophotometer (Thermo Fisher Scientific, Waltham, MA, USA) and was used to calculate the total RNA yield. Total RNA purity was assessed by measuring the A260/280 ratio. Samples with a total RNA yield of at least 1 μg and an A260/A280 ratio of at least 1.8 were considered of sufficient quality for further analysis [47].

### cDNA synthesis and quality assessment

Then, 1 μg of total RNA from each patient sample was converted to cDNA using the Transcriptor First Strand cDNA Synthesis Kit (Roche Applied Science, Mannheim, Germany) with a mix of random hexamer and oligo-dT priming in a 20 μl reaction volume.

In order to assess cDNA quality, before the amplifications of a set of genes on PCR arrays, *HPRT1* expression in each sample was measured. *HPRT1* is a well-known reference gene with low expression [48]; therefore, it is suitable for cDNA quality control. To estimate mRNA quality, 1 μl of cDNA was used to amplify the *HPRT1* gene in a 10 μl real time PCR reaction using a LightCycler 480 SYBR Green I Master (Roche). The specificity of target amplification was confirmed by melting curve analysis. On the basis of Cq for *HPRT1* assay, the sample was qualified or not qualified (Cq>35) for further analysis.

### Preamplification

The time-dependent fragmentation and small amount of RNA extracted from FFPE samples require a preliminary RNA amplification step to amplify RNA without altering the gene expression profile [49, 50]. In our experience and that of others [49, 51, 52] without the preamplification step, up to 40% of genes of interest (especially those with low expression) cannot be detected and reliably counted by real-time PCR in FFPE samples. Therefore, those cDNA samples that passed *HPRT1* quality control underwent preamplification using a RealTime ready cDNA Pre-Amp kit (Roche) according to the manufacturer's instructions. The pre-amplification temperature protocol consisted of 1 min at 95°C followed by 14 PCR cycles of denaturation at 95°C for 15 s and annealing/elongation at 60°C for 4 min.

### Real-time PCR arrays

Quantitative real-time PCR (qPCR) was performed on a Light-Cycler 480 II instrument (Roche) with real-time ready custom PCR Arrays (Roche) with pre-plated qPCR assays for 125 genes containing 120 targets and 5 reference genes in triplicate. Each reaction well at 10 μl final volume contained two primers and one Universal ProbeLibrary (UPL) probe, which was a short FAM-labeled hydrolysis probe containing locked nucleic acid (LNA). Special attention was paid to keep amplicons no longer than 100 bp to enhance detection sensitivity and reduce bias in the analysis of fragmented RNA isolated from FFPE tissue. Primers spanning an exon-exon junction were used (Supplementary Table S1). Quantitative real-time PCR was performed with the following temperature profile: pre-incubation at 95°C for 10 min and 45 cycles of amplification consisting of 95°C for 10 s, 60°C for 30 s and 72°C for 1 s.

### Analysis of real-time PCR arrays data

To identify the most stable reference genes across the study group, RefFinder [53], a web-based comprehensive tool (http://fulxie.0fees.us) was used to rank the analyzed reference genes: *GAPDH*, *RPLP0*, *SF3A1*, *B2M*, and *TBP*. The *GAPDH*, *RPLP0*, and *SF3A1* were identified as the most stable reference genes and used for normalization.

RT^2^ Profiler PCR Array Data analysis web-based software v. 3.5 (http://pcrdataanalysis.sabiosciences.com/pcr/arrayanalysis.php), which is dedicated to the data analysis of PCR arrays, was used. Any Cq value >35 was considered undetected. The ΔCq for the gene of interest was calculated by subtracting the geometric mean [48] of Cq for *GAPDH*, *RPLP0*, and *SF3A1* from the Cq for the gene of interest. Differences in expression between groups were calculated using the 2^−ΔΔCq^ method [54]. *P*-values were calculated based on two-tailed t-test, and *P*<0.05 was considered to indicate significance.

Search Tool for the Retrieval of Interacting Genes/Proteins v. 10 (STRING; http://string-db.org) linked to the Kyoto Encyclopedia of Genes and Genomes (KEGG) was used to recognize specific DNA repair pathways related to outcome that were significantly enriched and possible protein-protein interactions. A minimum of three counts and *P*<0.05 were considered to indicate the significant relevance of pathways.

## SUPPLEMENTARY MATERIALS TABLE




