# Supplementary material for: Pathological complete response after cisplatin neoadjuvant therapy is associated with the downregulation of DNA repair genes in BRCA1-associated triple-negative breast cancers
Source: Oncotarget. 2016 Sep 8;7(42):68662–73. doi: 10.18632/oncotarget.11900 (PMC5356581; doi:10.18632/oncotarget.11900)
Supplement: Supplementary file 1 [file oncotarget-07-68662-s001.pdf]

Pathological complete response after cisplatin neoadjuvant therapy is associated with the downregulation of DNA repair genes in *BRCA1*-associated triple-negative breast cancers

SUPPLEMENTARY TABLE

Supplementary Table S1: Genes and primers used in this study

See Supplementary File 1
