# Supplementary material for: Pathological complete response after cisplatin neoadjuvant therapy is associated with the downregulation of DNA repair genes in BRCA1-associated triple-negative breast cancers
Source: Oncotarget. 2016 Sep 8;7(42):68662–73. doi: 10.18632/oncotarget.11900 (PMC5356581; doi:10.18632/oncotarget.11900)
Supplement: Supplementary file 2 [file oncotarget-07-68662-s002.docx]

| Gene symbol | RefSeq | Forward Primer Sequence | Reverse Primer Sequence | UPL Probe Number | Assay ID |
| --- | --- | --- | --- | --- | --- |
| ABL1 | NM_007313 | AGAAGGACTACCGCATGGAG | GAGGGATTCCACTGCCAAC | 44" | 144473 |
| APEX1 | NM_001641 | CTCAAAGTTTCTTACGGCATAGG | GCTGTTACCAGCACAAACGA | 5" | 137234 |
| ATAD5 | NM_024857 | CCAAGTACTTTCTTCCGAGTGTC | CTTTTGGTTTCTTTATGGCTGAC | 75" | 144930 |
| ATM | NM_000051 | CCTGAATAACACACTGGTAGAAGATT | GGCTAAAATGTTTTTCAAACAGGTA | 146" | 102867 |
| ATR | NM_001184 | CGCTGAACTGTACGTGGAAA | CAATTAGTGCCTGGTGAACATC | 21" | 101358 |
| B2M | NM_004048 | CCGTGTGAACCATGTGACTTT | CCTCCATGATGCTGCTTACA | 117" | 102065 |
| BABAM1 | NM_014173 | CACAATGGCACTGAGGAGAA | GCCCATGAAGGCAAACATA | 63" | 144955 |
| BAP1 | NM_004656 | GGAAGATTTCGGTGTCAAGG | GAACAGGAAGATAAATCCATATACAGG | 25" | 102242 |
| BARD1 | NM_000465 | CCAAATGTTAAAGACCATGCTG | CTTCAGGTGCCCATGATTG | 163" | 114208 |
| BCCIP | NM_016567 | ACCTGAGGTGCTTGGAGGT | ACCAGGGCTGTCACTTGC | 35" | 101361 |
| BLM | NM_000057 | CCAAAAGCTGACTTCCTTGG | CTTCTGAGTCAGTCTTATCACCTGTC | 151" | 113359 |
| BRCA1 | NM_007294 | TCCAGAACAAAGCACATCAGA | AGACACCTCAAACTTGTCAGCA | 129" | 102885 |
| BRCA2 | NM_000059 | CCTGATGCCTGTACACCTCTT | GCAGGCCGAGTACTGTTAGC | 45" | 144646 |
| BRCC3 | NM_024332 | CATGGTCCACGAGACTTCTG | GGATTGGGATTTCGATTCTCT | 144" | 109515 |
| BRE | NM_004899 | TCCAGCTCTGCCTGACATC | TGGAACTGAGGGACAAAGGT | 55" | 100173 |
| BRIP1 | NM_032043 | TCCAAATGTGAAAGATCTACAGGT | CTGACGGCCAGGTAGAAGAC | 157" | 144984 |
| CDK12 | NM_016507 | GAACTCCCACAATGCCACA | CTCTGGTGGAAGAATGTGAGG | 16" | 109852 |
| CDK7 | NM_001799 | ACAGAGGCCTTAGAACAAGGAG | CTTTTTGGCTATTTCCCTCAGTAG | 47" | 101429 |
| CHEK1 | NM_001274 | CAACAAACCCCTCAAGAAAGG | TGGATTGAATGTGCTTAGAAAATC | 14" | 101443 |
| CHEK2 | NM_001005735 | TGAGGCTGCGGAGAGTGT | GACTCCCGAGACATCACGAC | 1" | 100283 |
| EMSY | NM_020193 | AATGTCGCTCCCAGCTTCT | GAGGATCCTTGGGTTATAATTGG | 39" | 116864 |
| ERCC1 | NM_001983 | AAGAGAAGATCTGGCCTTATGC | ACTTTCAAGAAGGGCTCGTG | 75" | 111288 |
| ERCC2 | NM_000400 | TCACCATGGCAACCTTCA | ATGGCCACCTGGTCATTG | 34" | 144922 |
| ERCC3 | NM_000122 | AAGGACTACAGGCTGCAAATG | GAAGGCTTCCAAGAAGATATGG | 161" | 100402 |
| ERCC4 | NM_005236 | CCACTGACACTCGGAAAGC | CACGCATATCCACAACTATGC | 72" | 115300 |
| ERCC5 | NM_000123 | CCAAGCGCAGAAGAACATTA | TTAAGCAAGCCTTTGAGTTGG | 82" | 113989 |
| ERCC6 | NM_000124 | CTGCGGAGAATGAAGTCAGAT | GCTCATCTGTAAGACGGCAAA | 140" | 114691 |
| ERCC8 | NM_000082 | ACACATGTAAAGCAGTGTGTTCC | CACACTGTATCTGTGAACATCAGG | 53" | 119312 |
| FAM175A | NM_139076 | GAAATATATTCCATGCTATCAGCTTTT | TTTCTTCAGTGCTTGCTCATTT | 124" | 143980 |
| FANCA | NM_001286167 | CACAGCCCTGTGCTGAAAG | CGCAAAGCTCCACTCTCTCT | 150" | 114091 |
| FANCB | NM_152633 | TTGAAGACAAACAAGAGAATCGTT | CCGTAATTCCCGAAAAGAAGA | 131" | 130616 |
| FANCC | NM_000136 | GGCAGCAGAGAGCACAGAC | GCTGCTGCTTCTGGACATT | 10" | 144910 |
| FANCD2 | XM_006713021 | CCCAGAACTGATCAACTCTCCT | CCATCATCACACGGAAGAAA | 69" | 114651 |
| FANCE | NM_021922 | TTGGTGTTGCAGTCACTCCT | GAGCTTCTCCATTAAGACACTGAA | 39" | 125211 |
| FANCF | NM_022725 | CTGCCCTGGAGACCTGTAAA | TCTGTCCAGATGCTAAGACCAG | 58" | 125882 |
| FANCG | NM_004629 | CCTGGCCTTGTTACTAGAGACC | AGTTTTCAGAAGTAACAGCAGATCC | 71" | 112023 |
| FANCI | NM_018193 | TGGCCAACAAGACAAGTGAT | TTTCTTGGTGGCTTTGGATAC | 130" | 138483 |
| FANCL | NM_018062 | ACCGTGTATGAGGGATTCATCT | GGCAACACTATCCTAAGGTGGA | 103" | 125220 |
| FANCM | NM_020937 | GCCACACCAGGTAGTGATATAAAG | TGGAGAATCTTCAGAACGAAGC | 113" | 144923 |
| FEN1 | NM_004111 | AACCCCGAACCAAGCTTTA | GGGCCACATCAGCAATTAGT | 82" | 111570 |
| FIGNL1 | NM_001042762 | CGGAGAGCAATCGTTTGAA | AAGTCCTAGCAGTGTTAGTCACCA | 83" | 144961 |
| GADD45A | NM_001924 | AGAGCAGAAGACCGAAAGGA | TGACTCAGGGCTTTGCTGA | 37" | 101471 |
| GAPDH | NM_002046 | AGCCACATCGCTCAGACAC | GCCCAATACGACCAAATCC | 60" | 141139 |
| GTF2H3 | NM_001516 | CCTGTGTTTTAGACTCCGACTCA | GGCACCTTCAGGTACAGTCC | 25" | 144913 |
| H2AFX | NM_002105 | AGCACTTGGTAACAGGCACA | CTCTGCCCTCCCCTAAATGT | 16" | 113494 |
| KAT5 | NM_006388 | GGTTTTTCCCCAGAATGGAG | TCAGAGCTGTCCTGGGAGTC | 20" | 101816 |
| LIG1 | NM_000234 | CTCTCCCATCTACCCTGCTG | TAAACCGAGGGAAGCGAAG | 138" | 137231 |
| LIG3 | NM_013975 | GCTGGCCACAAAGTCTTCTC | CCAGTGAAGATGTCCAGCAA | 7" | 114112 |
| LIG4 | NM_002312 | ACAGAGGTAACGGAGCTTGC | AAGGAACGTGAGATGCAACA | 147" | 115063 |
| MAPKAPK2 | NM_004759 | ACTCACTGACTTTGGCTTTGC | TCTCTGGACCCAGCACTTCT | 5" | 109820 |
| MDC1 | NM_014641 | GGGCGGCTACATATCTTTAGTG | CCGAGGTGTAGTGGGAAATC | 93" | 114472 |
| MGMT | NM_002412 | GTGATTTCTTACCAGCAATTAGCA | CTGCTGCAGACCACTCTGTG | 52" | 102265 |
| MLH1 | NM_000249 | TGCCAATTTTGGTGTTCTCA | AAGCATGGCAAGGTCAAAGA | 36" | 110983 |
| MMS22L | NM_198468 | CCAAGTGGGGTCAGAAGAAG | CATACCATAATCCTGGATAAACTGC | 33" | 144127 |
| MND1 | NM_032117 | GCAGATGTTCTTCACCCCTTT | AGATGGATGGATTTTTATTGTCCT | 136" | 135061 |
| MRE11A | NM_005591 | GGGAACGTCTGGGTAATTCTC | TCAAAACCTCCACTATAGTCCACTC | 106" | 101518 |
| MSH2 | NM_000251 | AATGTGTTTTACCCGGAGGA | TCAGAATTCCTCCTCTTTGAATTATC | 120" | 110812 |
| MSH6 | NM_000179 | TTTTTGATGACAGCCCAACA | CTGGGCTTCCTTTGATTTTG | 23" | 100622 |
| MUTYH | NM_012222 | CAGGAACAGCTCTTAGCCTCA | CTGTCCAGTGTTGGGAGCA | 25" | 143296 |
| NBN | NM_001024688 | TTTTGAATGCCAAACAGCAT | TCATTCTCTTCTGTTATCAACCTAGC | 10" | 101519 |
| NEIL1 | NM_024608 | GACCAAGCTGCAGAATCCAG | TCTCTGACCCGTAGCCTTTG | 67" | 113642 |
| OGG1 | NM_016821 | CTGCATCCTGCCTGGAGT | CCTGGGGCTTGTCTAGGG | 83" | 111525 |
| PALB2 | NM_024675 | CGTGGGTGTGATGCTGTACT | ACGTCACCTTCCAGGAACC | 37" | 113977 |
| PARG | NM_003631 | AACCCTTGACACGATTGCAT | CTTGCACAAGTCCTGCACTG | 10" | 144957 |
| PARP1 | NM_001618 | TGGAGGACGACAAGGAAAAC | TGTTGCTACCGATCACCGTA | 57" | 111143 |
| PARP2 | NM_005484 | GCATGCAATGAATTCTACACCA | AGTTCCTTCTGTGTCCGGATT | 25" | 125285 |
| PARP3 | NM_005485 | TTGGCAAGGGCATCTACTTT | CCCACACTTCATGCCAATAA | 150" | 130337 |
| PMS1 | NM_000534 | GAATGTAGACCTCGCAAAGTGAT | ATGGGTAATTGTCTGGATAGACG | 7" | 111489 |
| PMS2 | NM_000535 | CAGGTTTCATTTCACAATGCAC | AGACCTCATTCACGAGTCTGC | 12" | 137297 |
| PNKP | NM_007254 | TGGAGAAGTTGCTAGTGTTCACC | CGTCCCGTCCAGATCAAA | 37" | 105297 |
| POLB | NM_002690 | TGGCAGTTTCAGAAGAGGTG | TGGCTGTTTGGTTGATTCTG | 33" | 100748 |
| POLD3 | NM_006591 | CGCGTACTAAAATCTAAAACTTACCTG | GCAGGATTCACTCTCGTAGACTT | 60" | 142638 |
| POLH | NM_006502 | GCCATGATGCATTTACTGTCA | GTAGCACAGAGGAAAAGCATTG | 63" | 114292 |
| PPM1D | NM_003620 | ACCTGTGTGATGACTCCTTCC | CCATGGATCCTCCTCCAGT | 131" | 105326 |
| PRKDC | NM_001081640 | AAGGCGGCTTACCTGAGTG | CTGACATTTTTGTCAGCCAATC | 37" | 106448 |
| PSMC3IP | NM_013290 | GATGTGTTCGGGAACCTACAG | CAGCTGCTCCAGCGTCTT | 61" | 144916 |
| PTEN | NM_000314 | GCTACCTGTTAAAGAATCATCTGGA | CTGGCAGACCACAAACTGAG | 59" | 101040 |
| RAD21 | NM_006265 | CAGGGAGTTAAGCGAAAAGC | CCATCTGCTCTACCTGCTGA | 115" | 112344 |
| RAD23B | NM_002874 | GCAGCAACTACGACAGCAAC | CTGAGGCTGATTCCGTAAAAA | 65" | 114141 |
| RAD50 | NM_005732 | CCAGGAGCTCATAAAAGCTGA | TCCATTTTTAAGGTTTCTACATTGC | 111" | 110774 |
| RAD51 | NM_002875 | GGCGGTCAGAGATCATACAGA | AGATCCAGTCTCAATTCCACCT | 36" | 101531 |
| RAD51AP1 | NM_006479 | TCTAATTGCAGTGTAGCCAGTGA | GCTGCTTTTCTTTTCCCTTG | 119" | 144948 |
| RAD51B | NM_002877 | TGGCTTCTCAGGCAGACC | CCTAGTGCGGCTATCACACA | 78" | 112092 |
| RAD51C | NM_058216 | ATCTTTCATTGGGACCGAAA | CTTCTGGCTGGGTGACTTGTA | 145" | 127629 |
| RAD51D | NM_002878 | CACTGCCATCCTGTCCACT | CTGGGCCTCCTACAATTTCA | 54" | 143524 |
| RAD52 | NM_134424 | AAATAAGCTTCCACGCCAGTT | GGTCGGCAGCTGTTGTATCT | 25" | 115161 |
| RAD54B | NM_012415 | TCATGATCTGCTTGACTGTGAG | GAATTTTTCCAACGAATCACCT | 2" | 125942 |
| RAD54L | NM_003579 | GGCAACCTGGCCTAGTGAC | AACACTCCTGGATCTGGGTCT | 120" | 127608 |
| RBBP8 | NM_203291 | GCACACGTGTAAGGAATGTGA | GCGGAATCGGTGTCTTGA | 139" | 101541 |
| RECQL | NM_002907 | AGAATGGCGTCCGTTTCA | TTCTACTGCATGTAGCTCACTGG | 154" | 115393 |
| RECQL4 | NM_004260 | CCCTGCTGTCACTCATGGA | TCATGCCCGAGTGTATGC | 87" | 113815 |
| RECQL5 | NM_004259 | CTTGGACAGGAGGCTGATAAA | CCTCTCTAGTCCTGCAGTACACAAT | 71" | 125798 |
| REV1 | NM_016316 | CCTGAGTATAGAGGTCCCGTCA | CGGAGATCAGGTGGAAGTG | 33" | 137167 |
| RNF168 | NM_152617 | AGTGCAAGCTTAGAGCGTCTG | AGCAGACGAACTGGCTGATAG | 6" | 123757 |
| RPA1 | NM_002945 | CCAGTGCCCTATAATGAAGGA | CCATTCCCGAGCTTCCAT | 3" | 137309 |
| RPA2 | NM_002946 | GCCACCTGAGATCTTTTCAGA | CCAGAATATGTGTGGTGAACTCAT | 65" | 126299 |
| RPLP0 | NM_053275 | TCGACAATGGCAGCATCTAC | GCCAATCTGCAGACAGACAC | 6" | 101144 |
| SF3A1 | NM_005877 | GAGGTGTACGCACCAGGTC | ATGTCAGTACGCCGCTCAG | 108" | 122615 |
| SHFM1 | NM_006304 | AGAGGAAGACGACGAGTTTGA | CCCAATTATCCTCCCAGACA | 26" | 108040 |
| SLX4 | NM_032444 | AACCATCGCCAAACCTTTC | TGGCACTGCTGTTGTCAAA | 12" | 144951 |
| SMARCAL1 | NM_014140 | CAAGACGTGGAACTTCAGCA | GAGGCTCTGGGCTGCTTT | 122" | 126107 |
| SPIDR | NM_001080394 | CAGCTCAGGACACACAGACC | CTTCACCGAACATTCCACAG | 8" | 144919 |
| SSRP1 | NM_003146 | TGAGTCATTCAACCCAGGTG | AGGCGTTGCTGTCAAACTC | 69" | 138091 |
| STK11 | NM_000455 | AACCGGCCAAGAGGTTCT | GATGGGCACTGGTGCTTC | 88" | 106285 |
| SUMO1 | NM_003352 | AAGACAGGGTGTTCCAATGAA | CCCCGTTTGTTCCTGATAAA | 69" | 101560 |
| TBP | NM_003194 | GAACATCATGGATCAGAACAACA | ATAGGGATTCCGGGAGTCAT | 87" | 101145 |
| TDG | NM_003211 | ACCTACAGCTTGCCCAAGAG | TGCATGGATTTTCTCCGTAA | 157" | 103798 |
| TDP1 | NM_018319 | GCCAAGCCTTACGAGAACAT | TGTTCCAAACGCAATATCCA | 53" | 115468 |
| TOP3A | NM_004618 | AGTGGGTCGATGAGAACACC | TCTTCCTCTGTCTCCTGTCCA | 50" | 125825 |
| TOPBP1 | NM_007027 | TGCAGGACTGCTGGACACT | GTATGGAACTACTTCCCCATTCA | 4" | 113749 |
| TP53BP1 | NM_005657 | GGACAGAACCCGCAGATTT | CCTGTCTGACTGACCCCTTT | 24" | 114211 |
| UBA1 | NM_003334 | AGGGTGGAGAAATCATCGTTAC | GCAGCCGTGTCAGACTTTAAC | 163" | 137269 |
| UIMC1 | NM_016290 | GACCGGCCATTACTGGTG | TTTCTCCGTGGCATCCTTT | 165" | 103694 |
| UNG | NM_080911 | ACCCCCACACCAAGTCTTC | CCAGGATGACAACCTTCACA | 115" | 110850 |
| USP1 | NM_001017415 | GTGCTTTGCTGCTAGTGGTTT | GAGGTGTCAATAAAGGAGTGTTGA | 77" | 109303 |
| USP11 | NM_004651 | CCGCTTCTATAAGCTCTATCAGCTA | ATGCGACCTGACACCTCATA | 110" | 108324 |
| WRN | NM_000553 | TGCAGCCATTTCTTGTCAAA | GAAGGACAGTAGATGATTGTTGGA | 48" | 113170 |
| XPA | NM_000380 | GCAGCCCCAAAGATAATTGAC | TCGCATATTACATAATCAAATTCCA | 63" | 110783 |
| XPC | NM_004628 | TAAAGGGGTCCATGAGGACA | TGGCTGCAGATGTTATTTCG | 13" | 111265 |
| XRCC1 | NM_006297 | CTGGGACCGGGTCAAAAT | CAAGCCAAAGGGGGAGTC | 71" | 110721 |
| XRCC2 | NM_005431 | GCCTGTCAGCTTTTTACTGGA | CCTCAGAGTAGACTCCTGTAAGTTCA | 25" | 110795 |
| XRCC3 | NM_005432 | TGAGAACGGCCTCCTTACAC | GGAACCGCTCCTTCTGCT | 38" | 137302 |
| XRCC4 | NM_022550 | CTGATGGTCATTCAGCATGG | CCAACATATTTCCCTTTTTCCA | 123" | 114754 |
| XRCC5 | NM_021141 | TCCTTGAAAAACAGTAAGAAATATGC | GCTCATGGAGTCAATCAAAGC | 98" | 111044 |
| XRCC6 | NM_001469 | GCCCTCCCTGTTCGTGTA | CTTGATGAGCAGAGCACTGAA | 82" | 111420 |
